# Therapeutic effects of marshmallow (*Althaea officinalis* L.) extract on plasma biochemical parameters of common carp infected with *Aeromonas hydrophila*

**Published:** 2017-06-15

**Authors:** Mahdi Banaee, Vahid Soleimany, Behzad Nematdoost Haghi

**Affiliations:** *Department of Aquaculture, Faculty of Environment and Natural Resources, Behbahan Khatam Al-Anbia University of Technology, Behbahan, Iran*

**Keywords:** *Aeromonas hydrophila*, Biochemical parameters, Common carp, Marshmallow

## Abstract

This study evaluated preclinical and clinical safety of marshmallow (*Althaea officinalis* L.) extract as a naturopathic medicine in common carp deliberately infected with *Aeromonas hydrophila*. The fish were fed 0 (control), 2.50, 5.00 and 10.00 g of marshmallow extract for 60 days in a preclinical experiment and then, challenged with *A. hydrophila *for a 10-day experiment. Significant increases were observed in aspartate aminotransferase (AST), lactate dehydrogenase (LDH), alkaline phosphatase (ALP), creatine phosphokinase (CPK) activities and plasma creatinine levels in fish fed 10 g marshmallow extract per kg feed. However, alanine aminotransferase (ALT) significantly decreased on day 60. The fish fed 2.50 g marshmallow extract per kg feed indicated increased levels of total protein and globulin. There were no significant changes in albumin levels (*p *> 0.05). 2.50 and 5.00 g marshmallow significantly decreased triglyceride and cholesterol levels and increased glucose levels (*p *< 0.05). *A. hydrophila* significantly increased AST, ALT, LDH, ALP and CPK activities and plasma glucose, cholesterol, triglycerides and creatinine levels after 10 days (*p *< 0.05). Total plasma protein, albumin and globulin levels in fish challenged with *A. hydrophila *were significantly lower than the control group (*p *< 0.05). Marshmallow extract at 5.00 and 10.00 g can adjust plasma biochemical parameters in fish challenged with *A. hydrophila*. The results of preclinical studies and pharmaceutical toxicity of marshmallow extract revealed that dietary levels lower than 5.00 g were safe and effective. The results of this clinical study demonstrated that marshmallow extract (5.00 g kg^-1 ^feed) can protect fish against *A. hydrophila*.

## Introduction

Aeromonas infection is a serious problem in aquaculture and various antibiotics are used to treat or control fish morbidity. However, the overuse of antibiotics can lead to bacterial resistance and drug residues in final products and environment.^1^ Therefore, in recent years, many attempts have been made in veterinary science to replace chemically synthetic drugs with natural medicine.^2^^-^^4^ One of the most important aspects of veterinary pharmacology is identification and discovery of new drug compounds which can be divided into three phases including discovery, preclinical development and clinical trials. 

Medicinal plants have been widely used in folk veterinary medicine for treating or preventing from various diseases in farm animals.^5^^,^^6^ Herbal products provide an important source of potential medicines and often contain organic compounds with different effects including chemotherapeutic, immune-stimulant, bacteri-ostatic, bactericidal, antifungal and anti-parasitic functions.^3^^,^^7^^,^^8^ In the last two decades, numerous studies have been conducted to determine the feasibility of using herbal medicine in prevention and treatment of aquatic animals’ diseases.^2^^, ^^9^^-^^11^

Marshmallow (*Althaea officinalis*) is a medicinal plant, the roots, leaves and flowers of which are usually used in traditional medicine in many countries all over the world. This herb contains peptins, starch, monosaccharides, disaccharides, mucilage, flavonoids, anti-oxidants, coumarins, scopoletin, tannin, asparagines and many amino acids. The extracts obtained from the roots and flowers of marshmallow have antibacterial (both Gram-positive and Gram-negative bacteria), antifungal, anti-inflammatory, anti-mycobacterial and anti-cough properties^12^ as well as antiviral, anti-yeast, anti-complement^13^ and free radicals scavenging activities. The aqueous extract of marshmallow is also helpful in lowering hyperlipidemia, inflammation, reducing gastric ulcers and inhibiting platelets adhesion without showing any adverse effects on the consumers.^14^^,^^15^ This plant can also prevent inflammation through inhibition of cytokinins, interleukin-6 and tumor necrosis factor synthesis and/or release. The increases of phagocytic and macrophage activities^16^ and the number of T lymphocytes are the main immuno-modulatory effects of the marshmallow root extract. That is why marshmallow would be a good alternative in prevention and treatment of bacterial, viral and fungal infections of aquatic animals. 

Therefore, this study was carried out to determine the efficiency of marshmallow extract (*Althaea officinalis* L.) against experimental infection by *Aeromonas hydrophila*, so that marshmallow extract might eventually be developed as alternative controls for Aeromonas disease in aquaculture.

## Materials and Methods

**Fish preparation and storage conditions. **One hundred eighty common carps (average body weight and length: 37.65 ± 4.40 g and 14.15 ± 0.80 cm) were purchased from a private farm (Carp Farm, Shush, Iran) and transferred to the aquaculture laboratory of Aquaculture Department at Behbahan Khatam Al-Anbia University of Technology, Behbahan, Iran. After transferring, 15 fish were randomly allocated to each of 12 fiberglass tanks (300 L) equipped with aeration. Fish were acclimated to the experimental conditions for two weeks (24 ± 2 ˚C; pH: 7.40 ± 0.20; 16 L-8D; 40% water exchange rate per day). During acclimation, fish were fed a commercial diet (Beyza Feed Mill, Shiraz, Iran; including gross energy: 3500 Kcal kg^-1^; crude protein: 35 to 37%; crude lipid: 9 to 11%; crude fiber: 5%; moisture: < 10%; ash: < 10%; total volatile nitrogen: < 45 mg per 100 g) twice a day and near to 2% of their respective body weight.

**Extract of marshmallow flower. **The powder of dried flower of *A. officinalis* was mixed with distilled water and ethanol (1:1), and the mixture was put on the shaker for 24 hr at room temperature. The resulting hydro-alcoholic extract was filtered through Whatman filter paper and evaporated to dryness on a rotary evaporator until it became creamy and then, dried in an oven at 50 ˚C that finally gave 8 g (8% of initial amount) dried powder. The concentration used in the experiment was based on the dry weight of the extract (Table 1). 

**Fish diet preparation. **Experimental diets were prepared in the laboratory using powdered commercial feed (Beyza Feed Mill, Shiraz, Iran). In this study, prepared marshmallow flower extract was incorporated into basal diet. To enrich the normal diet, 2.50, 5.00 and 10.00 g of *A. officinalis* extracts were mixed with 1 kg feed. Each supplemented diet was mixed with distilled water (1 mL g^-1^) until a homogenous mixture was obtained. The dough was passed through a meat grinder and spaghetti strings were dried at 55 ˚C for 12 hr. Finally, they were crumbled and pellets with approximately 10 mm length were produced. The pellets were packed and stored at –20 ˚C until be used. The control diet was prepared by the same process, although no supplement was added.

**Bacterial culture.** *Aeromonas hydrophila *(MIC No: 1096; ATCC: 7965) was obtained from Pasteur Institute Iran.* A. hydrophila* was grown in nutrient broth (Oxoid Co. Ltd., Altrincham, UK) for 24 hr at 37 ˚C. The culture was centrifuged at 3000 *g* for 10 min at 4 ˚C, before the supernatants were discarded and the pellets were suspended in 0.90 % (w/v) saline. The bacterial suspensions were counted on hemocytometer slide at a magnification of 400× using a light microscope (model YJ 2005B; Nigbo Tianyu Optoelectronic Technology Co. Ltd., Ningbo, China).

**Table 1.** Plasma biochemical parameters of common carp fed various dietary levels of marshmallow extract (2.50, 5.00 and 10.00 g kg^-1^ feed) along with control group at the end of the preclinical stage. Data are presented as mean ± SD

**Blood biochemical parameters**	**Experimental groups**			
	**Control**	**2.50 g** **kg**^-1^	**5.00 g kg** ^-1^	**10.00 g** **kg**^-1^
**Aspartate aminotransferase (U L** ^-1^ **)**	50.50 ± 5.10^a^	61.43 ± 6.87^ab^	60.21 ± 14.31^ab^	63.85 ± 7.30^b^
**Alanine aminotransferase (U L** ^-1^ **)**	12.24 ± 4.12^b^	10.48 ± 1.41^b^	11.03 ± 0.90^b^	8.27 ± 2.82^a^
**Lactate dehydrogenase (U L** ^-1^ **)**	203.18 ± 38.00^a^	191.65 ± 33.35^a^	182.39 ± 22.71^a^	327.73 ± 15.24^b^
**Alkaline phosphatase (U L** ^-1^ **)**	66.05 ± 14.76^a^	86.14 ± 14.97^b^	70.43 ± 14.32^a^	84.52 ± 17.18^b^
**Creatine phosphokinase (U L** ^-1^ **)**	972.48 ± 317.13^a^	1013.57 ± 203.75^a^	1199.81 ± 155.99^ab^	1330.72 ± 438.19^b^
**Glucose (mg dL** ^-1^ **)**	66.48 ± 14.68^a^	60.49 ± 8.97^a^	80.34 ± 17.39^b^	102.53 ± 10.88^c^
**Total protein (mg dL** ^-1^ **)**	42.90 ± 4.90^a^	54.50 ± 6.80^b^	41.20 ± 11.60^a^	45.70 ± 2.30^a^
**Albumin (mg dL** ^-1^ **)**	23.40 ± 6.50^a^	25.90 ± 8.70^a^	24.40 ± 1.10^a^	22.00 ± 4.20^a^
**Globulins (mg dL** ^-1^ **)**	19.50 ± 6.40^a^	28.60 ± 14.00^b^	16.80 ± 12.80^a^	23.90 ± 4.80^ab^
**Cholesterol (mg dL** ^-1^ **)**	87.95 ± 9.93^b^	76.53 ± 8.95^a^	73.09 ± 8.63^a^	86.14 ± 6.58^b^
**Triglycerides (mg dL** ^-1^ **)**	236.64 ± 38.47^c^	202.69 ± 39.38^ab^	193.73 ± 17.65^a^	227.32 ± 48.35^bc^
**Creatinine (mg dL** ^-1^ **)**	0.40 ± 0.09^a^	0.39 ± 0.08^a^	0.46 ± 0.06^b^	0.36 ± 0.07^a^

abc Different superscripts indicate the signiﬁcant difference (*p* < 0.05, Duncan’s multiple comparison).

**The experimental design:** **Preclinical stage. **The preclinical experiment was done as a completely randomized design including three treatments and one control group in triplicates. The fish were fed the commercial diet supplemented by 0.00, 2.50, 5.00 and 10.00 g of the hydro-alcoholic extract of marshmallow flower. The fish were fed experimental diets twice a day and near to 2% of their respective body weight. After 60 days, nine fish were randomly captured from each group and anesthetized using clove powder solution (200 mg L^-1^). Then, blood samples were taken from caudal vein and stored in screw test capped tubes containing heparin as an anticoagulant at 4 ˚C. The samples were centrifuged for 10 min at 6000 *g*, at 4 ˚C. Plasma samples were immediately collected and stored –25 ˚C until biochemical analysis.^2^

**Clinical stage. **After 60 days of feeding experiment, the fish were challenged with *A. hydrophila* for 10 days. Challenge was by intraperitoneal injection with 0.10 mL of bacterial suspension containing ≈10^7 ^cells per mL. Mortalities were recorded for 10 days whereupon the survivors were examined pathologically.^17^ During the clinical experiment, fish were fed commercial diet enriched with 0.00, 2.50, 5.00 and 10.00 g of the hydro-alcoholic extract of marshmallow. At the end of the experimental period, survivals were taken and anesthetized as previously described. Blood samples were collected similar to the previous stage. The blood was centrifuged for 10 min at 6000 *g*, at 4 ˚C. Plasma was immediately collected and stored at –25 ˚C until biochemical analysis.

**Biochemical parameters of blood. **Biochemical analyses were carried out using commercial diagnostic kits supplied by Pars Azmun Company (Tehran, Iran) and a UV/VIS spectrophotometer (model Uni-co 2100; Unico Inc. Los Angles, USA). Total plasma protein content was measured by the Biuret method at 540 nm, albumin level was measured by the immediate bromcresol green reaction at 630 nm; plasma globulin content was determined via subtracting plasma albumin content from total protein concentration of samples,^18^ plasma glucose was measured by the glucose-oxidase method at 500 nm;^19^ plasma cholesterol levels were determined by cholesterol oxidase/peroxidase (CHOD-PAP) enzymatic method at 510 nm, triglyceride level was measured by glycerol-3-phosphate oxidase (GPO-PAP) enzymatic method at 546 nm^20^ and creatinine was measured by the JAFFE method at 510 nm.^21^ The activities of aspartate aminotransferase (AST) and alanine aminotransferase (ALT) of samples were assayed by NADPH consumption and its conversion to NAD^+^ at 340 nm, lactate dehydrogenase (LDH) activity was determined based on the conversion of pyruvate to lactate at 340 nm, alkaline phosphatase (ALP) activity was determined based on converting nitro phenol phosphate into nitrophenol and phosphate at 405 nm, creatinine phosphokinase (CK) activity was measured based on the conversion of creatinine phosphate into creatinine at 340 nm^22^ according to the instructions provided by the kit supplier.

**Data analysis. **One-way analysis of variance (ANOVA) was used to analyze the data. When ANOVA identified a significant difference among groups, Duncan’s multiple range tests were used to examine the significant differences among treatments using the SPSS (Version 19.0; IBM Corp., Armonk, USA) computer software. Significance of difference between control group and fish challenged with *A. hydrophila* was determined using *t*-Student test. Before the analysis, all the data were examined for normality (Shapiro-Wilk test). A *p-*value less than 0.05 was considered as statistically significance. Data are presented as mean ± SD. 

## Results

**Preclinical stage**. The activity of some biochemical parameters in the plasma of common carp on day 60 after being fed different supplemental levels of marshmallow extract is presented in Table 1.

Compared to the control, a significant increase was observed in plasma AST activity of fish fed marshmallow extract at 10 g kg^-1 ^feed. The results indicated that plasma ALT activity of fish fed marshmallow extract at 10 g kg^-1 ^feed showed a significant decrease on day 60 compared with the control group. Plasma LDH and CPK activities of the common carps which were fed 10 g kg^-1 ^feed significantly increased on day 60. Plasma ALP activity of fish fed marshmallow extract at 2.50 or 10 g kg^-1 ^feed significantly increased. 

The results indicated that plasma glucose levels in the fish fed marshmallow extract at 5.00 and 10.00 g kg^-1 ^feed showed a considerable increase on day 60 compared with the control group. Plasma total protein level in the fish fed marshmallow extract at 2.50 g kg^-1 ^feed was significantly higher than that of control group on day 60. No significant changes were found in the albumin level on day 60. Oral administration of 2.50 g marshmallow extract per kg feed significantly increased plasma globulin level on day 60. Plasma cholesterol and triglyceride levels of fish fed marshmallow extract at 2.50 and 5.00 g kg^-1 ^feed showed a significant decrease on day 60 when compared with the control. Plasma creatinine level of fish fed marshmallow extract at 5.00 g kg^-1 ^feed showed a significant increase on day 60.

**Fig. 1:**
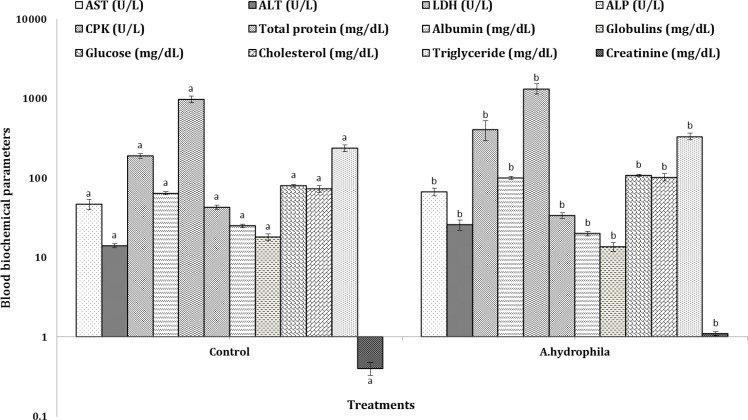
Blood biochemical parameters of common carps (*C. carpio*) infected with *A. hydrophila*.

^ab^ Different superscripts indicate the signiﬁcant difference (*p* < 0.05, *t*-Student test).

**Fig. 2:**
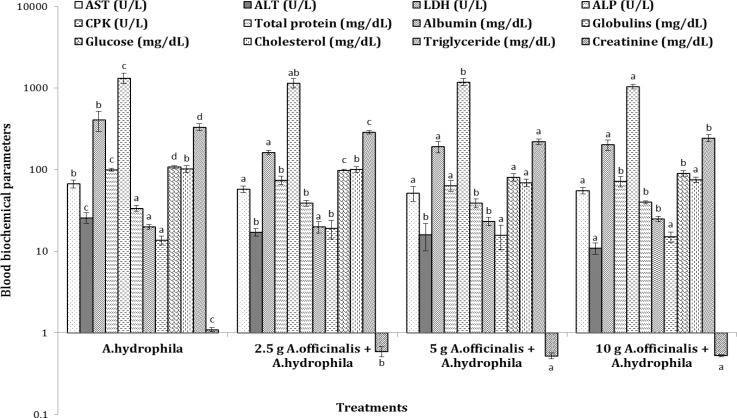
Blood biochemical parameters of common carps (*C. carpio*) fed with *A. officinalis *extract-enriched diets against *A. hydrophila*.

^abcd^ Different superscripts indicate the signiﬁcant difference (*p *< 0.05, Duncan’s multiple comparison).

**Fig. 3:**
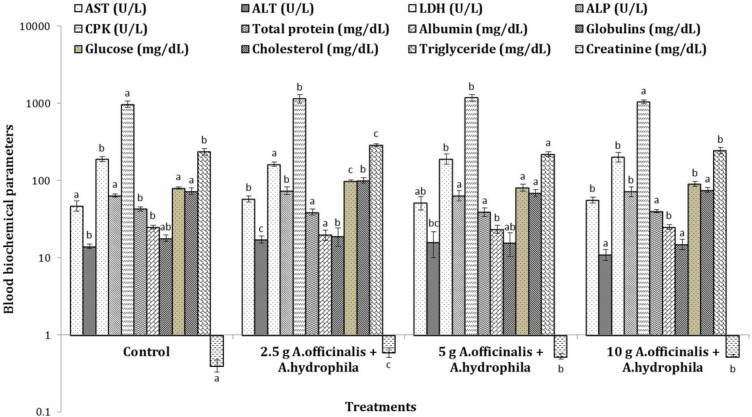
Blood biochemical parameters of common carps (*C. carpio*) fed with *A. officinalis *extract-enriched diets against *A. hydrophila* as compared with the control group.

^ab^ Different superscripts indicate the signiﬁcant difference (*p* < 0.05, Duncan’s multiple comparison).

**Clinical stage. **Changes observed in plasma biochemical parameters of common carp fed varied concentrations of marshmallow extract and simultaneously infected with *Aeromonas hydrophila *are provided in Figures 1 to 3.

The AST, ALT, LDH, ALP and CPK activities in plasma of fish infected with *A. hydrophila* were significantly higher than those in control group. Plasma total protein, albumin and globulins of fish decreased after being challenged with *A. hydrophila* (*p* < 0.05). The results indicated that plasma glucose, cholesterol, triglycerides and creatinine levels of fish challenged with *A. hydrophila* were significantly higher than those of the control group (*p *< 0.05, Fig. 1). 

There was a significant increase in AST, ALT, LDH, ALP and CPK activities of fish challenged with *A. hydrophila* (*p *< 0.05), whereas their activities decreased in fish fed a diet enriched with marshmallow extract as compared to the group challenged with *A. hydrophila*. Glucose, triglycerides and creatinine levels significantly increased in fish with no supplemental feeding and challenged with *A. hydrophila*. However, plasma glucose, triglycerides and creatinine levels significantly decreased in fish fed marshmallow extract-enriched diet. Dietary marshmallow extracts significantly elevated levels of total protein concentrations in the plasma of fish infected with *A. hydrophila* compared to the untreated-challenged group. Feeding infected fish marshmallow extract at 5.00 and 10.00 g kg^-1 ^feed increased albumin levels. Plasma globulin levels were significantly increased in fish fed marshmallow extract-enriched diet (at 5.00 g kg^-1 ^feed) as compared to the group challenged with *A. hydrophila*. When fish were challenged with *A. hydrophila* and fed 5.00 g marshmallow extract per kg feed, a decrease in plasma cholesterol level was observed (Fig. 2).

The results showed that administration of marsh-mallow extract at 5 g kg^-1 ^feed resulted in normal activities of the AST and ALP in fish challenged with *A. hydrophila*. Oral administration of marshmallow extract (10.00 g kg^-1 ^feed) returned plasma ALT and CPK of fish challenged with *A. hydrophila* to normal state (Fig. 3). Plasma glucose levels of fish challenged with *A. hydrophila* and treated with marshmallow extract at 5.00 g kg^-1 ^feed restored to normal (Fig. 3). In this study, plasma total protein levels in infected fish fed marshmallow extract were significantly lower than those in the control group. However, albumin and cholesterol levels in fish treated with *A. hydrophila* and fed at 5.00 and 10.00 g marshmallow extract per kg feed returned to normal (Fig. 3). Administration of marsh-mallow extract returned plasma globulins to a normal state in fish challenged with *A. hydrophila*. Also, oral administration of marshmallow extract (10 g kg^-1 ^feed) returned plasma triglyceride levels in fish challenged with *A. hydrophila* to normal. However, plasma creatinine levels of fish challenged with *A. hydrophila* and treated with marshmallow extract were significantly higher than those of the control group (Fig. 3).

## Discussion

Active compounds of herbal products can positivity affect varied physiological functions in animals. Therefore, certain herbs have pharmaceutical effects and the most important biochemical effects of herbs and their derivatives can be attributed to adaptogens, alkaloids, antioxidants, micronutrients, minerals, saponins and polysaccharides present in the plants. 

Our results indicated a significant increase in AST, LDH, ALP and CPK activities in the fish fed 10.00 g marshmallow extract may be due to the cellular toxicity. Furfural is one of the compounds in marshmallow extract which turns into the toxic pyromucic acid in liver after oxidation.^23^ Therefore, furfural may indirectly cause oxidative stress and damage the liver cells.^24^ Increased activity of AST, ALT and ALP is reported in the rats treated with furfural during seven days.^24^ The results showed that 10.00 g marsh-mallow treatment significantly decreases ALT activity.

Alterations in plasma glucose levels in the fish indicate the effects of compounds present in marshmallow extract on the mechanisms controlling the uptake, storage and metabolism of glucose. Increased levels of blood glucose on day 60 in the fish fed marshmallow extract at 5.00 and 10 g kg^-1 ^feed may indicate cellular toxicity caused by compounds such as furfural in this plant. Same findings have been reported when rats fed furfural.^24^

Plasma protein is the major extracellular protein which is synthesized by liver cells. Since there is a close relationship between the rate of hepatic protein synthesis and total protein concentration in plasma,^25^ the increased concentration of total protein in plasma of the fish fed marshmallow extract at 2.50 g kg^-1 ^feed indicates increased protein synthesis in liver. It has been reported that materials such as thymol and eugenol prevent protein breakdown.^26^ Also, a significant increase in plasma total protein was reported in the fish fed with mistletoe (*Viscum album*) extract, stinging nettle (*Urtica dioica*), ginger (*Zingiber officinale*),^27^ silymarin (*Silybum marianum*)^25^ and yarrow (*Achillea millefolium *L.).^28^

No significant changes were observed in albumin levels in preclinical study. A significant increase in plasma globulin in the fish fed 2.50 g marshmallow extract per kg feed improves the fish immune system. Also, increases of albumin and globulin were reported in the fish fed with silymarin.^25^

Decreased cholesterol and triglyceride levels in the fish fed marshmallow extract at 2.50 and 5.00 g kg^-1 ^feed could be due to the effects of marshmallow compounds on the synthesis, uptake and metabolism of cholesterol. It was found that carvacrol administration leads to decreased cholesterol and triglyceride levels in the plasma of rats treated with D-galactosamine.^29^^,^^30^ The inhibitory effects of bioflavonoids on HMG-CoA reductase activity, which has a great role in cholesterol biosynthesis, can account for decreased cholesterol level in the fish fed with marshmallow extract-enriched diet. Moreover, the presence of soluble fibers can decrease total cholesterol in plasma.^31^ Further, the fiber in the fish diet can provide the grounds for removing cholesterol from digestive system through liver stimulation for bile acids synthesis.^32^

Compounds such as pectin and mucilage in marsh-mallow extract can not only increase the secretion of bile acids, but also reduce cholesterol uptake in intestine.^33^ The presence of fatty acids in marshmallow extract including linoleic and oleic acids is effective in LDL lipoprotein and cholesterol reductions.^27^^,^^33^

In the present study, a significant increase was found in the plasma creatinine level on day 60 in the fish fed with 10.00 g marshmallow extract. Thus, this change may be associated with renal damages in fish. 

Changes in blood biochemical parameters are the main clinical symptoms were observed in fish infected with *A. hydrophila* in this study. Our results indicated a significant increase in AST, ALT, LDH, ALP and CPK activities in fish challenged with *A. hydrophila* as compared to controls (*p *< 0.05). Thus, the increase in these enzyme activities in the present study may be related to an increase in the generation of reactive oxygen species in fish challenged with *A. hydrophila.*^34^ Marshmallow extract has a mixture of natural terpenes and terpenoids.^23^ These compounds including carvacrol, thymol, linalool, beta-bisabolene and terpinene have numerous therapeutic properties such as antioxidant activities.^35^^-^^37^ Thus, administration of marshmallow extract may prevent oxidative stress. In fact, the results of this study indicated that marshmallow extract concentrations of 5.00 g or more could improve cell membrane function in varied tissues. So, it may prevent leakage of intracellular enzymes into the blood. Oral administration of thymol and carvacrol activates antioxidant enzymes and protects hepatocytes against severe damages.^38^ Carvacrol supplement reduced AST, ALT and LDH activities in plasma of the fish treated with D-glucosamine.^39^

Increase in blood glucose of fish challenged with *A. hydrophila* may reflect an increased energy requirement to neutralize the toxin secreted by *A. hydrophila*. Administration of 5.00 g marshmallow extract significantly normalized glucose levels in fish challenged with *A. hydrophila. *Regulation of blood glucose in fish may be partly due to induction of insulin secretion or may be attributed to the activation of glycogen synthesis and a healthy liver function. Flavonoids in the plants may reduce glucose uptake in intestine via inhibition of sodium-dependent glucose transport.^40^ Moreover, it has been suggested that carvacrol significantly reduces glucose level by improving insulin resistance in diabetic rats. ^41^

Malnutrition, reduced efficiency of liver in protein synthesis and reduction of nutrient absorption, especially protein, in the digestive system may be important factors in decreasing plasma total protein. Decreases in plasma albumin and globulin levels of fish challenged with *A. hydrophila* may be due to reduction in total protein levels in plasma. Decreased globulin levels may reduce the resistance of fish to pathogens. Albumin concentration is fairly high in blood and it acts as the carrier of many drugs and hormones. The increase of albumin level may play a role in transferring the compounds present in the marshmallow extract to the target tissues of fish challenged with *A. hydrophila*. Amino acids present in the *A. officinalis* extract may increase protein synthesis in the liver and other tissues.^23^ Increased levels of globulins in fish fed with marshmallow extract may increase resistance to *A. hydrophila* infection. 

The increase of blood triglyceride and cholesterol levels may be due to liver dysfunction in fish challenged with *A. hydrophila*. Destruction of cell membranes can also lead to increased levels of cholesterol in plasma. Triglyceride uptake impairment by adipose tissue may also increase triglycerides. Previous studies have shown that linalool found in marshmallow extracts has certain effects on the metabolism of triglycerides and oral administration of marshmallow extracts may decrease triglyceride concentration in blood of fish.^23^ Moreover, phytosterols can decrease blood cholesterol levels by interfering with the absorption of cholesterol from intestine.^42^ Therefore, the restoration of cholesterol and triglyceride levels at normal ranges in the blood of fish fed marshmallow extract at 5.00 and 10.00 g kg^-1 ^feed and simultaneously infected with *A. hydrophila* at the end of the experiment may be attributed to the influence of carvacrol and phytosterols on the biosynthesis and absorption of cholesterol and triglycerides in liver and intestine, respectively. A significant increase found in plasma creatinine level of fish infected with *A. hydrophila* may be attributed to renal dysfunction and/or damage. Administration of 10 g marshmallow extract significantly returned creatinine to normal level in fish challenged with *A. hydrophila.*

The results of preclinical experiment showed that using marshmallow extract supplemented diet (less than 5.00 g kg^-1 ^feed) is safe. In clinical experiment, our results showed that feeding common carps with dietary supplement, i.e. 2.50, 5.00 and 10.00 g marshmallow per kg feed, may lead to considerable resistance against *A. hydrophila* infection. Marshmallow extract at 5.00 g kg^-1 ^feed leads to a higher protection after challenge compared with the other concentrations of marshmallow extract. 
